# The intrinsic role and mechanism of tumor expressed-CD38 on lung adenocarcinoma progression

**DOI:** 10.1038/s41419-021-03968-2

**Published:** 2021-07-05

**Authors:** Long Gao, Yuan Liu, Xiaohong Du, Sai Ma, Minmin Ge, Haijun Tang, Chenfeng Han, Xin Zhao, Yanbin Liu, Yun Shao, Zhao Wu, Lianjun Zhang, Fang Meng, F. Xiao-Feng Qin

**Affiliations:** 1 Institute of Systems Medicine, Chinese Academy of Medical Sciences and Peking Union Medical College, Beijing, 100005 China; 2 Suzhou Institute of Systems Medicine, Suzhou, 215123 Jiangsu China; 3 Institute of Clinical Medicine Research, the Affiliated Suzhou Hospital of Nanjing Medical University; Suzhou Science and Technology Town Hospital, Suzhou, China

**Keywords:** Cancer therapy, Lung cancer

## Abstract

It has been recently reported that CD38 expressed on tumor cells of multiple murine and human origins could be upregulated in response to PD-L1 antibody therapy, which led to dysfunction of tumor-infiltrating CD8^+^ T immune cells due to increasing the production of adenosine. However, the role of tumor expressed-CD38 on neoplastic formation and progression remains elusive. In the present study, we aimed to delineate the molecular and biochemical function of the tumor-associated CD38 in lung adenocarcinoma progression. Our clinical data showed that the upregulation of tumor-originated CD38 was correlated with poor survival of lung cancer patients. Using multiple in vitro assays we found that the enzymatic activity of tumor expressed-CD38 facilitated lung cancer cell migration, proliferation, colony formation, and tumor development. Consistently, our in vivo results showed that inhibition of the enzymatic activity or antagonizing the enzymatic product of CD38 resulted in the similar inhibition of tumor proliferation and metastasis as CD38 gene knock-out or mutation. At biochemical level, we further identified that cADPR, the mainly hydrolytic product of CD38, was responsible for inducing the opening of TRPM2 iron channel leading to the influx of intracellular Ca^2+^ and then led to increasing levels of NRF2 while decreasing expression of KEAP1 in lung cancer cells. These findings suggested that malignant lung cancer cells were capable of using cADPR catalyzed by CD38 to facilitate tumor progression, and blocking the enzymatic activity of CD38 could be represented as an important strategy for preventing tumor progression.

## Introduction

Studies have demonstrated that CD38 is one of the hallmarks of immune cells, which expressed on variety of immune cells such as B cells, NK cells, T cells, and macrophages, and involved in regulating the differentiation, activation, and tolerance of immune cells [1, 2]. And the FDA has approved two CD38 antibodies (Daratumumab and Isatuximab) for the treatment of multiple myeloma due to the promising preclinical and clinical activities of CD38 in hematopoietic system [3, 4]. More recently, Chen L uncovered a novel mechanism that tumor expressed CD38 was upregulated in tumor-bearing mice that responding to PD-L1 antibody, which led to immune tolerance and resistance. And it needs to be further addressed whether PD-L1 combined with CD38 antibody treatment could overcome PD-L1 resistance in pre-clinical or clinical settings [5]. Indeed, the clinical outcomes of lung cancer patients treated with combining PD-L1 and CD38 antibody are not satisfactory [6]. Thus, intrinsic mechanisms for the role of CD38 in the solid tumor warrant further exploration.

Researches have proved that the elevation of CD38 expression plays an inverse role in the regulation of nicotinamide adenine dinucleotide (NAD^+^) and maintenance of NAD^+^ homeostasis is critical for maintaining the normal function of the immune cells, such as cytotoxic (CD8+) T cells and macrophages [7, 8]. CD38 possesses NAD^+^ glycohydrolase activity and ADP ribosyl cyclase activity, both of which could convert NAD^+^ to cADPR as well as hydrolase activity that converted cADPR to ADPR [9, 10]. The formation of cADPR and ADPR catalyzed by CD38 from NAD^+^ are dominated by a series of amino acid residues especially Cysteine123 and Cysteine205 (for mouse) as well as glutamate146 and glutamate226 (for human) [11, 12]. Both ADPR and cADPR are important second messengers via ryanodine receptors (RyRs) to mediate the transmembrane transport of Ca^2+^ from endoplasmic reticulum in hematopoietic cells [13, 14]. In addition, the level and enzymatic activity of CD38 gradually comes to increase during aging and leads to NAD^+^ decline. Inhibiting the function of CD38 ameliorates age-related metabolic dysfunction [15].

But it is controversial about the role of tumor expressed CD38 and its enzymatic activity in the non-hematogenic tumor environment. A study had reported that CD38^lo^ prostate cancer cells exhibited stem-like characteristics and surrounded with inflammatory cells in the glands, which inducing cancerous transformation of prostate cells and negatively correlated with prognosis [16]. In contrast, Bu X suggested that knocking out of CD38 in lung cancer cells resulted in an upregulation of NAD^+^, which led to increasing CD8 + T cell infiltration and predicted a better prognosis [17]. Orazio Fortunato identified that CD133+CXCR4+ lung cancer stem cells were able to initiate distant metastasis and led to a resistance for chemotherapy. They also found that CD133+CXCR4+ cancer stem cells evaded immune surveillance via increasing expression of CD38 and CD73 [18]. Understanding the essential mechanisms of CD38 employed by tumor cells in tumor microenvironment represents an attractive therapeutic opportunity to selectively target tumor cells.

Herein, we aimed to investigate whether tumor expressed CD38 could affect the proliferation, progression, and metastasis in vitro and in vivo. The hypothesis of this study was that the enzymatic activities of CD38 was essential in mediating cell survival and metastasis in tumor microenvironment. Furthermore, we hoped to find that blockade enzymatic activity or competitive inhibiting cADPR could be efficacious to counter the tumor.

## Materials and methods

### Cell culture and transfection

A549, MDA-MB-231, PNAC-1, PanCO2, SW480, HCT116, 293T, BEL7402 and HepG2 cells were cultured in DMEM while LLC cells were maintained in RPMI-1640. The small molecule inhibitors used in this work were seen in additional Table 1. siRNAs targeting *TRPM2* (Genepharma, China) was carried out according to the instructions (seen in additional Table 2). CD38 and TRPM2 sgRNA were colonized into Lenti-V2, pX458 plasmid and then transfect into cells according to the manufacturer’s protocol. The Lentiviral-FG-EF/HTLV vector was used for constructing CD38-over-expression after packaged in 293T cells using FuGENE (Promega, E2311) transfection. CD38-cysteine205 and cysteine123 mutation were performed with QuickMutation™gene mutagenesis kit (Beyotime, D0206). Similar methods to get human CD38 mutation (glutamate146 and glutamate226 mutation).

**Table 1:** Clinical characteristics of the 158 NSCLC patients.

Characteristics	Number		Results		*p*-value
			Positive	Negative	
Age (years)		156			0.2002
<60		105	82	23	
>60		53	35	18	
Gender		158			0.5075
Male		78	53	27	
Female		78	47	31	
Adenocarcinoma stage		135			0.0947
Poorly		41	23	18	
Moderately		51	30	21	
advanced		43	33	10	
TNM stage		156			0.0059**
I		42	15	27	
II		40	12	28	
III		34	15	19	
IV		40	26	14	
Distant metastasis		149			1.001
Yes		39	26	13	
No		110	72	38	
Chemotherapy		64			1.000
Yes		16	9	7	
No		48	26	22	

*P < 0.05; **P < 0.01; ***P < 0.001

### HPLC analysis

HPLC was conducted on Agilent 1290 infinity liquid chromatograph Plus C18 column (2.1 × 50 mm 1.8 μm), Acchrom X-Amide column (4.6 × 250 mm 5μm), eluting with a linear gradient of 0–80% CH_3_CN in triethylammonium acetate buffer within 30 min at the rate of 1000 μL/min. The concentration of each substance and the mixed standard was dissolved in pure water. The injection volume was 1 μL. Specific details will be provided if request.

### Animal studies

LLC cells were either injected into subcutaneous of female C57BL/6 mice at 2 × 10^5^/mouse. Tumor growth was measured every two days (volume = (length × width^2^)/2). And 5 × 10^6^ of A549 cells were subcutaneously injected into the right oxter of *BALB/c nude* mice and tumor growth was monitored once per weeks. In addition, C57BL/6 mice injected with 2 × 10^5^ of LLC cells and treated with PBS (Control), a single dose of 78c (i.p., 15 mg/kg, at day 7) or 8-Br-cADPR (i.p., 500 μg/kg, at day 7, every two days). All groups would be sacrificed and the weight of tumor also be recorded.

### Intracellular Ca^2+^ measurements

LLC and A549 tumor cells were digested and re-suspended in Hanks buffer containing 1% FBS with permeable Ca^2+^ indicator Fluo-3 AM (1 μM, BIOSS). The intracellular Ca^2+^ changes and average fluorescence intensity were measured by the flow cytometer and analyzed by FlowJo software.

### Flow cytometry assays

For apoptosis assays, Annexin V and PI staining was performed according to the manufacturer’s instructions. For tumor-infiltrating immunocytes, tumor tissues were prepared as single cell suspension and blocked with anti-mouse CD16/32 (BD), and then added concurrently with antibodies (seen in additional Table 4). Analysis was performed through FlowJo software and using single-color compensation as controls.

### cADPR measurements

The detection of cADPR from cell lines, cancerous and non-cancerous pleural effusion was carried out according to the instructions of the Amplite™ Fluorimetric cADP-Ribose Assay Kit (Cat.20305). In brief, cells in logarithmic phase were separated by “40:40:20 with 0.1 M formic acid based approach”. prepared and added cADPR standards and test samples (50 µL), and incubated for 1 h after adding ADRPC working solution (50 µL); then added 40 µL Quest Fluor™ NAD Probe plus 40 µL Assay Solution and continued incubation for another 20 min. Finally added 30 µL enhancer solution and incubated for another 20 min. Monitor fluorescence intensity detected at Ex/Em = 420/480 nm by Synergy plate reader (BioTek). Specific details will be provided if request.

### Transwell assays

A549 and LLC cells were digested and appended into the upper chamber in serum-free medium. 20% FBS medium was added to the downer-chamber, and related small molecular inhibitors were also mixed if necessary. Cells in the supreme chambers were removed and the other side of the chambers were fixed with 4% paraformaldehyde and then stained with 0.1% crystal violet and photographed at bright field microscope.

### Colony formation assays

Cells were seeded at the density of 500 cells/well into 24-well plate and cultured for seven days. Cells were fixed with 4% paraformaldehyde and stained with 0.1% crystal violet solution, followed by image capture at bright field microscope.

### RNA-sequences and Real-time quantitative PCR assays

For RNA-sequences, total RNAs from A549 cell lines (CD38 WT and KO) were extracted using the TRIzol (Invitrogen), according to the supplier’s recommendations. mRNA expression profiling was performed accordance with the protocol described by the Illumina manufacturer (GenePharma, Suzhou, China). Additional analyses were performed using Bioconductor package Limma. For qRT-PCR, RNA were extracted by Trizol kit and reversed into cDNA solution. Quantitative PCR was designed by gene-specific primers and GAPDH as reference gene (seen in additional Table 5). Samples were then analyzed for mRNA expression via qRT-PCR by using the LightCycler 480 (Roche) instrument and the relative expression of mRNA was normalized to the matched GAPDH.

### Western blotting assays

Cells were lysed in RIPA lysis buffer and denatured in SDS loading buffer for total proteins. After denaturation, equal protein was separated by 10% SDS-PAGE and incubated with antibodies against CD38 (ThermoFisher, 1:500), KEAP-1 (Proteintech, 1:2000), NRF2 (CST, 1:2000), β-ACTIN (Proteintech,1:2500), GAPDH (Proteintech, 1:2000), TRPM2 (Abcam, 1:1500). Secondary antibodies were incubated for 1 h at room temperature, and visualized by the ECL substrate.

### Cell viability assays

CCK-8 kits were used to estimate cell proliferation assays. Tumor cells were seeded in 96-well plates overnight then treated with different concentration of cADPR, 8-br-cAPDR, 2-APB, and ML-385 for 48 h. And absorption at 450 nm was detected with a Synergy plate reader (BioTek).

### Immunohistochemistry assays and clinical data collection

Tissues chips were purchased from Seville Biology Company (Wuhan, china) with 3 μm sections. Chips underwent dewaxing, re-hydration, antigen retrieval, blocking, and then incubated with CD38 (ThermoFisher, 1:50) antibodies overnight. Sections were incubated with secondary antibody, washed and developed with DAB. Pictures were obtained using a Nikon microscope camera and NIS-Elements software and with a digital whole-slide scanner (Leica, SCN400F).

### Human clinical data analysis

Genomic data of human specimens for subjects with Non-small-cell lung carcinoma were obtained from Kaplan–Meier Plotter (https://kmplot.com), the TNM plot database (https://www.tnmplot.com/) and GEPIA (http://gepia.cancer-pku.cn). Kaplan–Meier survival analysis was conducted for evaluating the patient prognosis according to their correlation expression of CD38 and TRPM2 signature under alternative condition. Data were collected using empirical cumulative distribution function (ECDF) plots.

### Quantification and statistical analysis

All data were presented as mean and error bars represent standard error of mean (SEM) from at least two or three biological replicates. Unpaired two-tailed parametric *t*-test and Chi-square were calculated for statistical analyses. For survival analysis, the data were plotted and compared using the log-rank test. Pearson’s correlation analysis was used to assess the correlation between genes. All statistical analysis was done using GraphPad Prism (v.6.0).

## Results

### The upregulation of CD38 was associated with tumor progression and predicted a poor outcome

**Fig. 1:**
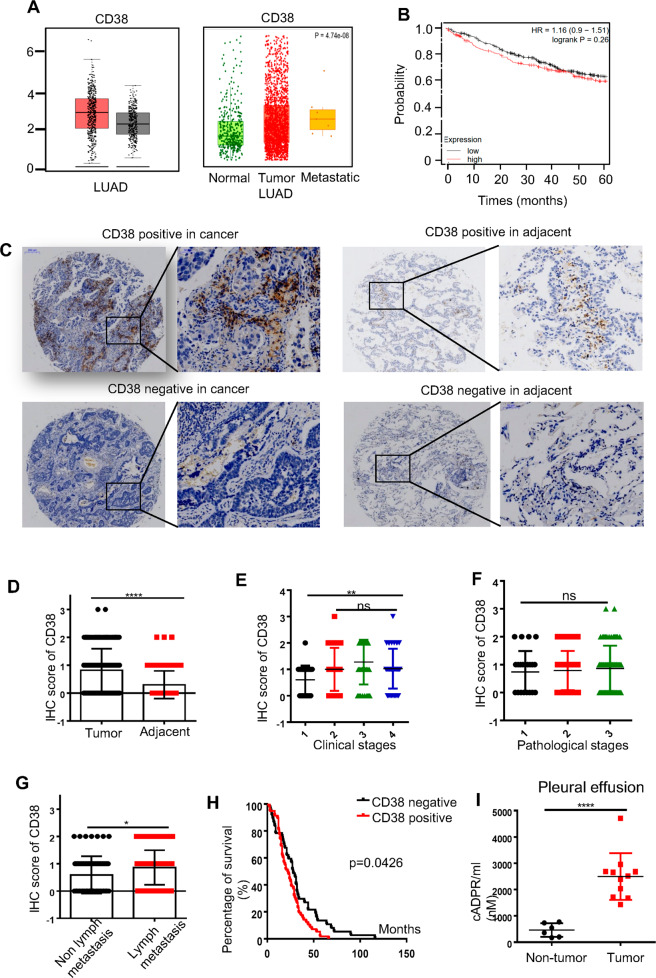
The expression of CD38 in tumor microenvironment predicted a poor outcome. **A** Public data GEPIA and TNM plot database were used to analysis of the significant expression of CD38 in lung normal tissues, lung adenocarcinoma, and metastatic lung adenocarcinoma. **B** Kaplan–Meier Plotter database was selected to analysis the prognosis of CD38 in lung adenocarcinoma. **C** IHC staining for CD38 were performed from the lung adenocarcinoma and adjacent normal patient tissue microarray (*n* = 158). Representative images of cell staining intensity were shown. **D** Statistical analysis of the expression of CD38 in cancer specimens compared with adjacent normal was shown. **E**–**G** The percentage of CD38 positive cells were analyzed and used to generate an IHC-score for each sample that passed quality control. Statistical analysis of the relation of clinical stage (**E**), pathological stage (**F**), lymph metastasis (**G**) with CD38 expression in tumor specimens was shown. **H** Correlation of CD38 expression with the overall survival was performed by Kaplan–Meier OS analysis. **I** The pleural effusion from patients suffer with adenocarcinoma and non-tumorous control were detected by Amplite™ Fluorimetric cADP-Ribose Assay Kit to measure cADPR. *P* values were calculated with ANOVA test. *P* values were calculated with ANOVA test, ns, no significant difference; *P < 0.05; **P < 0.01; ***P < 0.001.

It remained unknown whether the expression of tumor CD38 was associated with the prognosis of lung adenocarcinoma patients. Here we employed tissue microarrays of lung adenocarcinoma specimens to analyze CD38 expression by immunohistochemically staining (Seville Biology Company (Wuhan, China), Chort No.1601). We identified that the expression of CD38 was related to a potentiality of metastasis but a shortened survival in lung adenocarcinoma (Fig. 1A, B). Among the 158 paired adjacent and cancer tissue microarrays, we found that 62% of cancer case exhibited positive staining for CD38, while only 28.3% were positive for adjacent tissue (Fig. 1C, D, *p* < 0.001). The levels of CD38 in advanced tumors was significantly higher than that in early-stage (Fig. 1E, Table 1), but not associated with pathological characteristics (Fig. 1F). Our clinical data also demonstrated that CD38 was predicted a poor prognosis and promoted the metastasis (Fig. 1G, H). Then we collected a small scale of cancer-pleural effusion and non-cancerous-pleural effusion from volunteer patients, and found that the concentration of cADPR in cancerous pleural effusion was significantly higher than that of non-cancerous patients (Fig. 1I). We also confirmed that CD38 was mainly responsible for the production of cADPR in malignant pleural effusion, since no obvious CD157 was detected (Fig. S1). Taken together, these results indicated that CD38 revealed a poor survival of the lung adenocarcinoma patients.

### The enzymatic activity of CD38 was critical for lung cancer progression

We initially confirmed that CD38 was highly expressed on the surface of A549 and LLC cells (Fig. S2A). To clarify the roles of CD38 in lung cancer, we constructed CD38 knocked out (KO) cells, CD38 mutant cells (A549-CD38 and LLC-CD38 mutant cell, MU) (Fig. S2A–C) [11, 12], and CD38 overexpressed cells (OE) (Fig. S2A–C). We also transfected a red fluorescent protein in LLC cells (LLC-T2), which would visualize tumor metastasis in vivo. No significant difference of tumor growth was observed between LLC-T2 and LLC in the mice model (Fig. S2D). We further illustrated that CD38 was certainly expressed on multiple tumor cell lines across human and murine species (Fig. S2E).

**Fig. 2:**
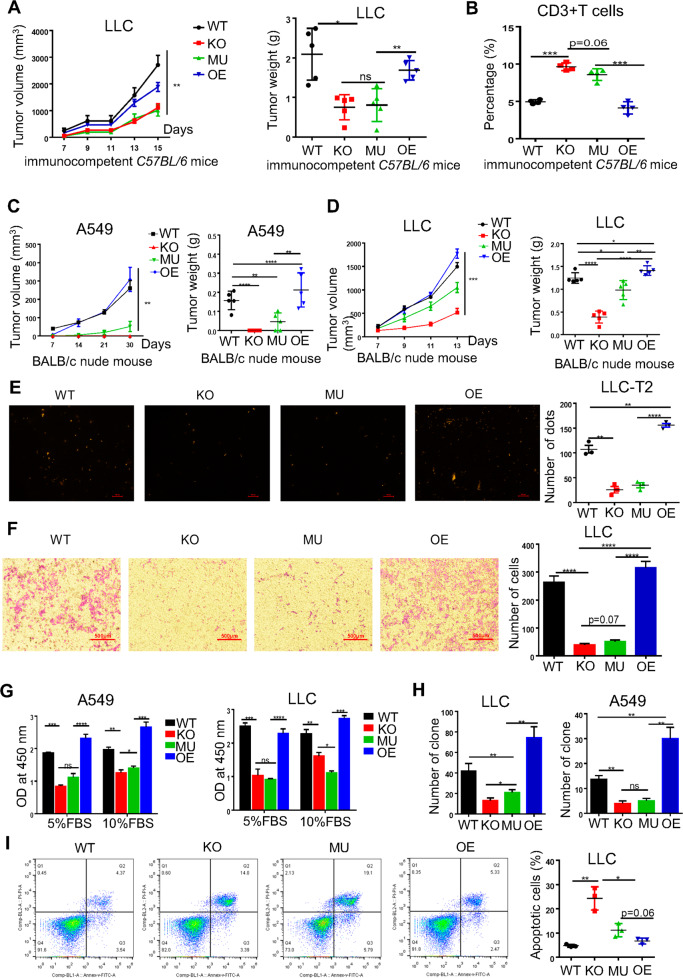
The enzymatic activity of CD38 was vital for cell proliferation, migration, and tumor progression. **A** LLC cells were separately implanted into immunocompetent C57BL/6 mouse (left). Tumors were measured every two days at day of 7. The tumor growth curve was shown with tumor sizes (*n* = 5). Mice were sacrificed at day of 15 after injection. The primary tumor mass is shown on the right (right). **B** FACS analysis showed the proportion of CD3 T+ tumor infiltrating lymphocyte (TIL) cells from primary tumors in immunocompetent mice (*n* = 5). **C** Growth of subcutaneous A549 tumors (3 × 10^6^ cells/mouse) in immune-deficiency BALB/c-nude mice (*n* = 5) (left). Tumors were measured every week at beginning on day 7. Mice were sacrificed at day of 30 after injection. The primary tumor mass was shown on the right (right). **D** 1.2 × 10^5^ of LLC tumor cells were implanted into BALB/c-nude mice (*n* = 5). The tumor growth curve was shown with tumor sizes (left). LLC tumorigenesis monitored every one day. Representative tumor mass was recorded after sacrificed at day of 13 after injection (right). **E** Number of spontaneous lung metastases in LLC-T2 tumor-bearing immunocompetent C57BL/6 mouse was shown. The visual numbers were randomly chosen in each group (*n* = 3), calculated by image J software. **F** LLC (1.5 × 10^5^ cells per well) were seeded at the upper-chambers for 24 h and then the numbers of migrated cells that adhered to the lower surface of the trans-well chambers were counted under microscope randomly chosen visual fields per well within the area (*n* = 3). **G** LLC and A549 cells were seeded at a density of 3000 cells/well into 96-well plates for 48 h and then cell viability was determined using a CCK-8 assay, and the results were shown as the OD value to represent the relative proliferation ability of the cells (*n* = 3). **H** A549 cells were cultured at a density of 500 cells/well into 24-well plates for 7 days and then the number of colony formation was visualized by staining with 0.1% crystal violet, and the results were shown as the number of positive area randomly (*n* = 3). **I** Representative FACS plots showing the apoptotic of LLC with different level of CD38 expression (*n* = 3). ANOVA or *t* test was used to analyze the data. The data were presented as mean ± SEM, ns, no significant difference; *P < 0.05; **P < 0.01; ***P < 0.001, ****P < 0.0001.

In immunocompetent mouse, we found that CD38 MU and KO tumor cells exhibited the impaired capability of tumor growth, mass and lung metastasis compared with CD38 WT and OE group (Fig. 2A, E). An increasing proportion of tumor-infiltrated CD3^+^ T cells was observed in CD38 MU and KO group while macrophages or myeloid-derived suppressor cells (MDSCs) showed no differences among all the groups (Fig. 2B, Fig. S2F). Similar results were also obtained that growth of CD38 KO and MU cells was significantly reduced in size and mass compared with WT and OE in BALB/c-nude and CD38 deficiency mouse (Fig. 2C, D, Fig. S2G). In CD38 deficiency mouse, a rising number of CD3^+^ T cells were also observed in CD38 MU and KO group (Fig. S2H). Consist with precious studies, we also demonstrated that tumor growth was dramatically inhibited in the CD38 deficiency mice in comparison with that of control WT mice (Fig. S2G, H).

In our vitro experiments, we observed that CD38 KO and MU cells exhibited an inhibition of cell proliferation, colony formation and migration but an increasing rate of apoptosis in both A549 and LLC lung cancer cells, and the overexpression of CD38 could rescue the ability of cell survival (Fig. 2F–I, Fig. S2I, J). Collectively, our data showed that the enzymatic activity of CD38 was required for tumor progression.

### CD38 utilized its hydrolytic product cADPR to promote tumor development

**Fig. 3:**
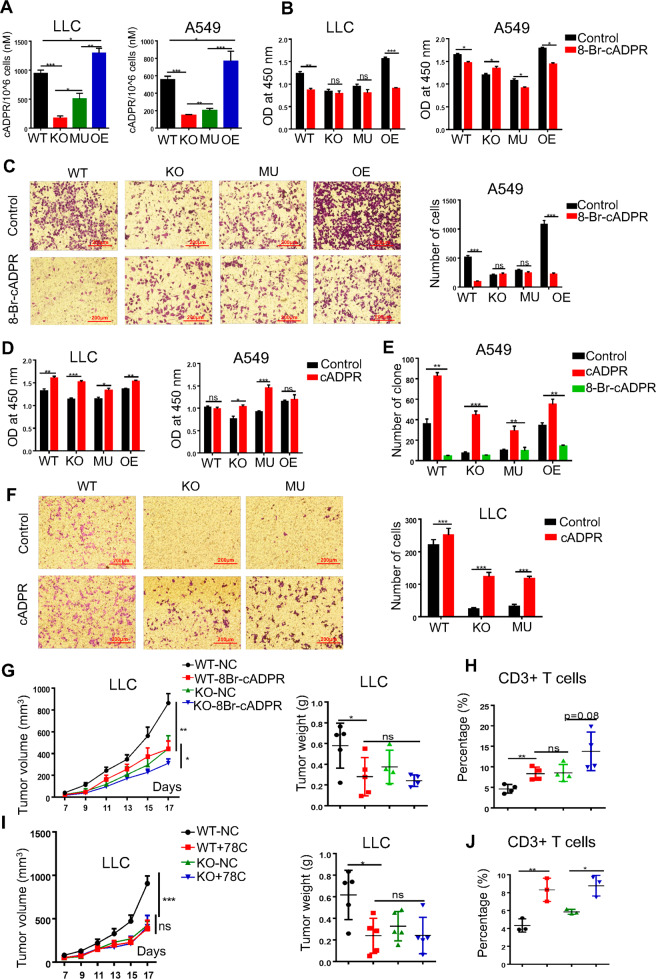
CD38 used its hydrolyze product cADPR to promote tumor survival. **A** A549 and LLC tumor cells were subjected to Amplite™ Fluorimetric cADP-Ribose Assay Kit to measure cADPR. The results were shown as the relative concentration of cADPR (*n* = 3). **B** LLC and A549 cells were seeded at a density 3000 cells/well into 96-well plates treated with 8-Br-cADPR (10 μM for LLC, 20 μM for A549) for 48 h and then the cell viability was detected using a CCK-8 assay, and the results were represented as the OD value (*n* = 3). **C** A549 (1.0 × 10^5^/well) cells were seeded at the upper-chambers adding with 8-Br-cADPR (20 μM for A549) for 24 h and then the numbers of migrated cells that adhered to the lower surface of the trans-well chambers were counted under an inverted microscope randomly chosen visual fields per well within the area (*n* = 3). **D** LLC and A549 cells were seeded at a density of 3000 cells/well into 96-well plates administrated with cADPR (50 nM for LLC, 100 nM for A549) for 48 h and then cell viability was detected using a CCK-8 assay, and the results were expressed as the OD value (*n* = 3). **E** A549 cells were cultured at a density of 500 cells/well into 24-well plates treated with either 8-Br-cADPR (20 μM for A549) or cADPR (100 nM for A549) for 7 days and then the number of colony formation was visualized by staining with 0.1% crystal violet, and the results were shown as the number of positive cells randomly (*n* = 3). **F** LLC (1.5 × 10^5^/well) cells were seeded at the upper-chambers adding with administrated with cADPR (50 nM) for 24 h and then the numbers of migrated cells that adhered to the lower surface of the trans-well chambers were counted under an inverted microscope randomly chosen visual fields per well within the area (*n* = 3). **G** LLC cells were separately implanted into immunocompetent C57BL/6 mouse models (2.0 × 10^5^/mouse). 8-Br-cADPR (500 μM/kg) or PBS was injected into mice (intraperitoneal) twice a week for 2 weeks beginning on day 5 after tumor cells implanted (left). Tumors were measured every two days at day of 7. Mice were sacrificed at day of 18 after injection. The tumor mass was shown with tumor sizes (right). **H** FACS analysis showed the proportion of CD3 T+ tumor infiltrating lymphocytes (TILs) cells from primary tumors in immunocompetent C57BL/6 mice (*n* = 5). **I** LLC cells were separately implanted into immunocompetent C57BL/6 mouse models (2.0 × 10^5^/mouse). 78C (10 mg/kg) or a PBS control was injected into mice (intraperitoneal) twice a week for 2 weeks beginning on day 5 after LLC tumor cells were subcutaneously implanted (left). Tumors were measured every two days at day of 7. Mice were sacrificed at day of 18 after injection. The tumor mass was shown with tumor sizes (right). **J** FACS analysis showed the proportion of CD3 T+ tumor infiltrating lymphocytes (TILs) cells from primary tumors in immunocompetent C57BL/6 mice (*n* = 5). Data were shown as mean ± SEM. *P* values were calculated with ANOVA or *t* test. ns, no significant difference; *P < 0.05; **P < 0.01; ***P < 0.001, ***P < 0.001.

As described previously, CD38 was well known as a major NAD glycohydrolase and participated in the generation of adenosine (ADO) and our results showed that the enzymatic activity of CD38 was important for tumor progression. We thus investigated the changes of NAD^+^ and adenosine (ADO) affected by CD38 enzymatic activity. Compared with CD38 WT cells, CD38 KO or MU cells led to an upregulation of NAD^+^ but a declination of ADO (Fig. S3A). But we did not observe any significant changes of proliferation and migration in tumor cells after treated with NAD^+^ precursor NMN or ADO (Fig. S3B, C), indicated that CD38 might regulate tumor cell proliferation and migration independent on ADO and NAD^+^. We further showed that CD38 KO and MU cells displayed remarkably downregulated level of cADPR compared with WT and OE cells (Fig. 3A). And administration of 8-Br-cADPR (a competitive CD38 enzyme hydrolysate inhibitor) could result in a significant inhibition of cell proliferation, colony formation, and migration on CD38 WT and OE tumor cells, but not on CD38 KO and MU cells (Fig. 3B, C, E, Fig. S3D). Furthermore, cADPR could rescue the cell proliferation, colony formation, and migration in the CD38 KO and MU tumor cells (Fig. 3D–F, Fig. S3E). These results suggested that CD38-cADPR signal was actually involved in regulation of cells progression. We then examined the therapeutic effects of 8-Br-cADPR in LLC-bearing immunocompetent mice. It was found that both 8-Br-cADPR and 78C (an enzyme inhibitor of CD38) could significantly inhibit tumor growth and tumor mass in CD38 WT-LLC-tumor-bearing mice while no therapeutic effects were observed in CD38 KO mice (Fig. 3G). Similarly, we also found that 8-Br-cADPR and 78C could increase the infiltration of CD3+ T cells (Fig. 3H–J, Fig. S3F, G). In summary, our results demonstrated that CD38 mediated tumor cell progression through CD38-cADPR signaling and blockade this axis might potentially serve as a novel therapeutic intervention for lung cancer.

### cADPR could promote the concentration of cytoplasmic Ca^2+^

**Fig. 4:**
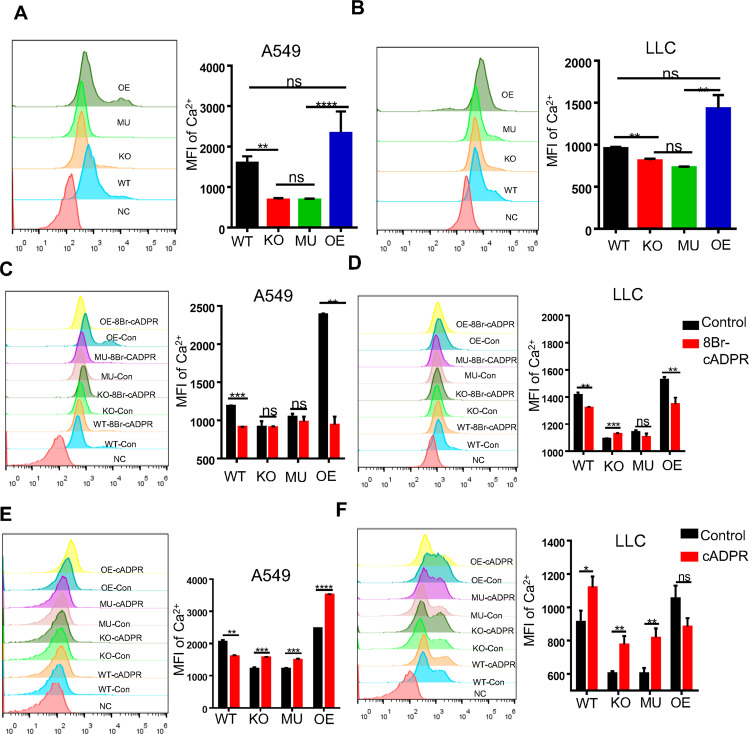
cADPR promoted the concentration of cytoplasmic Ca^2 +^. **A**, **B** FACS analysis was used to detect the MFI of cytoplasm Ca^2+^ in A549 (**A**, left) and LLC (**B**, right) tumor cells (*n* = 3). **C**, **D** FACS analysis showed the MFI of cytoplasm Ca^2+^ in A549 (**C**, left) and LLC (D, right) tumor cells treated with 8-Br-cADPR (for A549 was 20 μM, for LLC was 10 μM) for 1 h (*n* = 3). **E, F** FACS analysis showed the MFI of cytoplasm Ca^2+^ in A549 (**E**, left) and LLC (**F**, right) tumor cells added with cADPR ((for A549 was 100 nM, for LLC was 50 nM) for 1 h) for 0.5 h (*n* = 3). Data were shown as mean ± SEM. ANOVA or test was used to analyze data. ns, no significant difference; *P < 0.05; **P < 0.01; ***P < 0.001, ****P < 0.0001.

It was reported that cADPR could modulate intracellular calcium in immune cells via the activation of RyRs anchored on the endoplasmic reticulum. Ca^2+^ is a dynamic second messenger induced by many factors, leading to pleiotropic effects on cell survival including changes of migration, proliferation, and metabolism. Thus, it is not surprising that aberrant regulation of Ca^2+^ signals can lead to pathological phenotypes, including cancer progression [19]. However, given the highly context-specific nature of Ca^2+^ dependent changes in tumor cell function, its role in the TME needs to be further explored. Here we confirmed that CD38 KO and MU cells exhibited a lower concentration of cytoplasmic calcium than that of WT cells, while an increasing level of calcium were shown in CD38 OE cells (Fig. 4A, B). Exogenously administration of 8-Br-cADPR was found to induce a significantly decrease of the cytoplasmic calcium in CD38 WT and OE tumor cells but not in CD38 KO and MU cells (Fig. 4C, D). When stimulated with a minimal level of cADPR, the cytoplasmic calcium of CD38 KO and MU cells was rescued, and cADPR could further upregulate the level of calcium in WT and OE cells (Fig. 4E, F). Herein, our results demonstrated that CD38 might use cADPR to influence cytoplasmic calcium concentration of tumor cells.

### cADPR induced the concentration of intracellular Ca^2+^ via TRPM2 channel

**Fig. 5:**
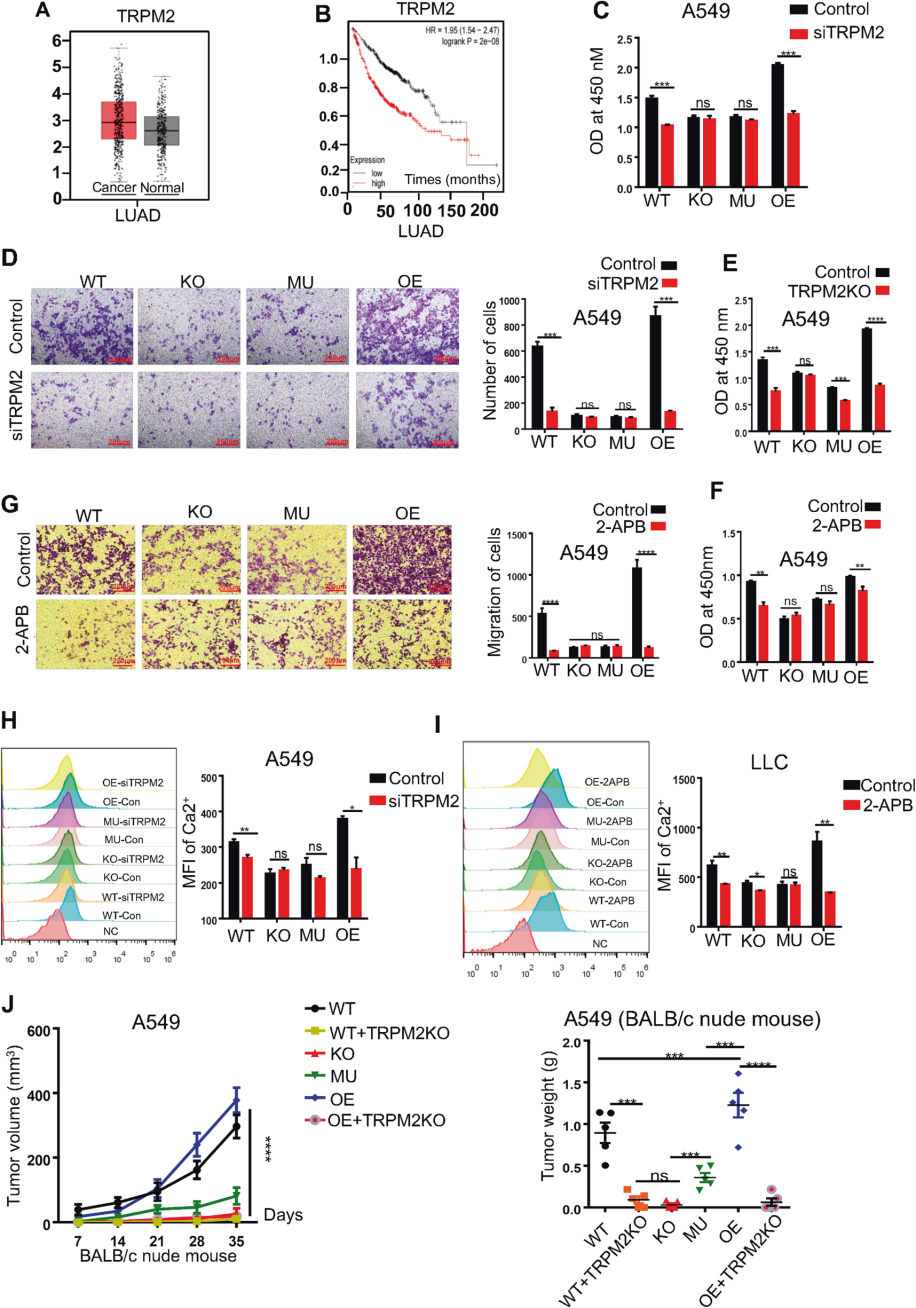
cADPR induced the concentration of intracellular Ca^2+^ via TRPM2 channel. **A** GEPIA public database was used to assess the expression of TRPM2 in the lung adenocarcinoma compared with adjacent tissues. **B** Kaplan–Meier plotter database was used to perform the overall survival for lung adenocarcinoma tissue microarray with different TRPM2 levels. **C** A scramble control (TRPM2-con) or TRPM2 knock down (siTRPM2) A549 cells were seeded at a density of 3,000 cells/well into 96-well plates for 48 h and then cell viability was detected by using a CCK-8 assay, and the results were expressed as the OD value (*n* = 3). **D** A scramble control and TRPM2 knock down (siTRPM2) A549 (1 × 10^5^/well) cells were seeded at the upper-chambers adding for 24 h and then the numbers of migrated cells that adhered to the lower surface of the trans-well chambers were counted under an inverted microscope randomly chosen visual fields per well within the area (*n* = 3). **E** TRPM2-control or TRPM2-KO A549 tumor cells were seeded at a density of 3000 cells/well into 96-well plates for 48 h and then cell viability was detected using a CCK-8 assay, and the results were expressed as the OD value (*n* = 3). **F** Control (TRPM2-con) or small molecular inhibitor (2-APB, 200 μM) A549 cells were seeded at a density of 3000 cells/well into 96-well plates for 48 h and then cell viability was detected using a CCK-8 assay, and the results were expressed as the OD value (*n* = 3). **G** Control or a small molecular inhibitor (2-APB, 200 μM) A549 cells (1 × 10^5^/well) were seeded at the upper-chambers adding for 24 h and then the numbers of migrated cells that adhered to the lower surface of the trans-well chambers were counted under an inverted microscope randomly chosen visual fields per well within the area (*n* = 3). **H** FACS analysis showed the MFI of cytoplasm Ca^2+^ in A549 cells with a scramble control (TRPM2-con) or TRPM2 knock down (siTRPM2) (*n* = 3). **I** FACS analysis was used to perform the MFI of cytoplasmic Ca^2+^ in LLC cells with control or a small molecular inhibitor (2-APB,100 μM, 1 h) (*n* = 3). **J** Growth of subcutaneous A549 tumors (3 × 10^6^ cells per mouse) in immune-deficiency BALB/c-nude mice was performed (*n* = 5) (left). Tumors were measured every 2 days beginning on day 7 after injection. Mice were sacrificed at day of 35 after injection. The primary tumor mass was also shown on the right (right). Data were presented as mean ± SEM. ANOVA or test was used to analyze data. ns, no significant difference; *P < 0.05; **P < 0.01; ***P < 0.001, ****P < 0.001.

We then hypothesized that cADPR might regulate the influx of extracellular calcium by activating TRPM2, among which its NUDT9H domain could be activated by cADPR [20]. Using GEPIA database, we found that the expression of TRPM2 in tumor was higher than that of normal tissues (Fig. 5A) and predicted a poor prognosis of lung adenocarcinoma patients (Fig. 5B). Next, it was confirmed that TRPM2 was expressed both in A549 and LLC cells (Fig. S4A). Along with the down-regulation of TRPM2 by siRNA in A549 cells (Fig. S4B), the inhibition of cell proliferation and migration was mainly occurred in CD38 WT and OE cells (Fig. 5C, D). When further knocking out TRPM2 in A549 tumor cells (Fig. S4C), similar results were also observed (Fig. 5E). Furthermore, 2-APB exhibited similar affection on CD38 WT and OE cells (Fig. 5F, G, Fig. S4D). This phenomenon might be explained by which the loss of CD38 enzyme activity resulted in the lack of cADPR and the inactivation of TRPM2 channel.

Along with inhibition of TRPM2 by siRNA or 2-APB, the decreasing concentration of calcium was observed in CD38 WT and OE tumor cell than that of control (Fig. 5H, I). Our results showed that all of TRPM2-deficient A549 tumor cells exhibited a significant reduction of tumor burden compared with that of control but independent on the expression of CD38 (Fig. 5J). Thus, we speculated that CD38-cADPR signal at least in partly through the activation of TRPM2 to promote the tumor survival. Further confirming the expression of TRPM2 in a variety of human cell lines (Fig. S4E), we found that cADPR could promote cell proliferation and migration, while 8-Br-cADPR shown on the contrary (Fig. S4F, G). Altogether, our results strongly suggested that the generation of cADPR by CD38 via TRPM2 channel to promote the cell progression.

### cADPR regulated KEAP1/NRF2 signal pathway to facilitate tumor progression

**Fig. 6:**
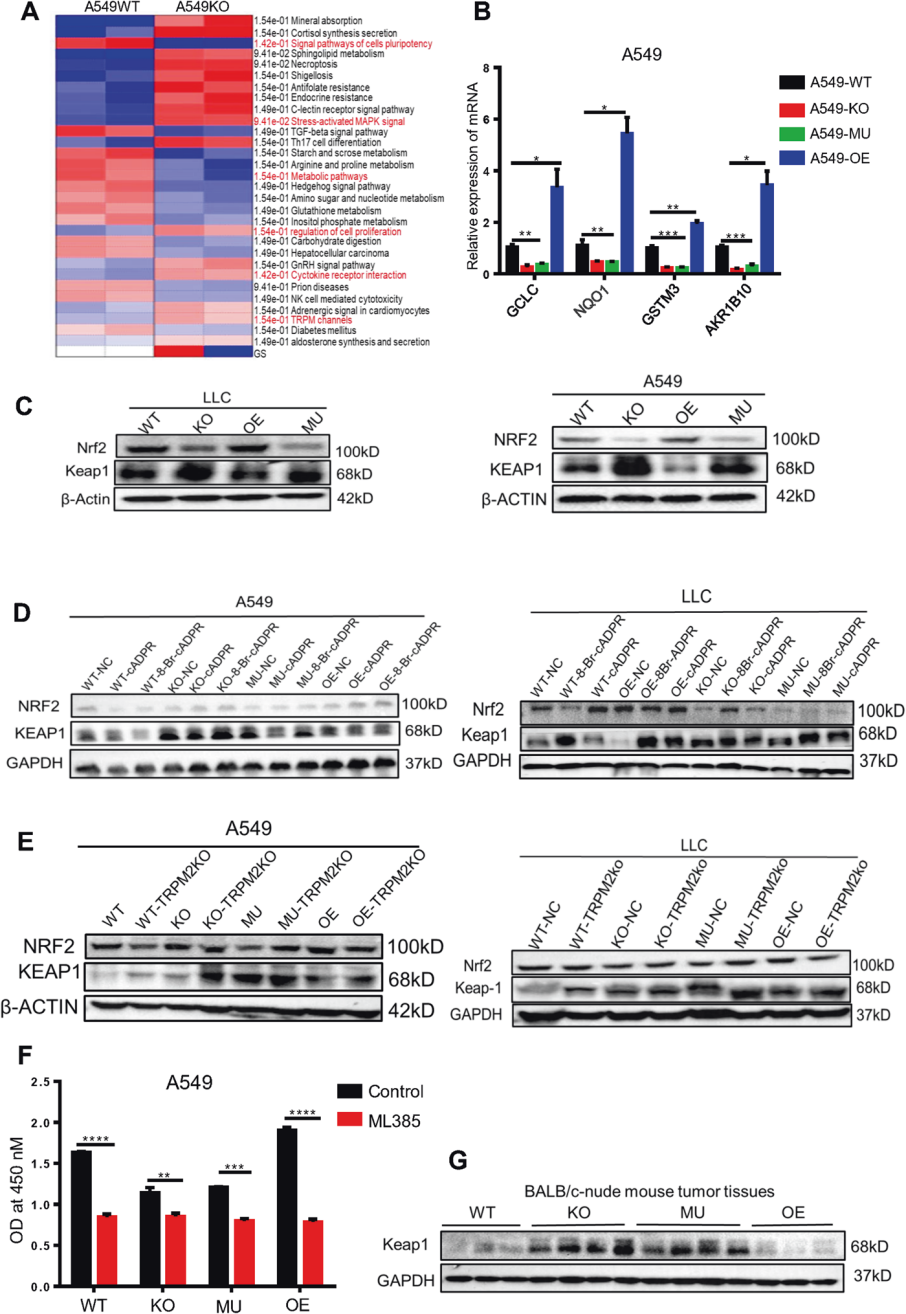
cADPR regulated KEAP1/NRF2 signal pathway to facilitate tumor progression. **A** Heat map showed the significant differences of signaling pathways from A549 CD38 WT and CD38 KO tumor cells by Kyoto Encyclopedia of Genes and Genomes (KEGG) analysis. **B** Relative mRNA expression levels of GCLC, NQO1, GSTM3, and ARK1B10 were shown in A549 cells calculated by qRT-PCR. And GAPDH was used as internal control (*n* = 3). **C** Western blotting was confirmed the expression of KEAP1 and NRF2 in A549 and LLC cells. GAPDH was shown as control. **D** Western blotting was used to detect the expression of KEAP1 and NRF2 in A549 and LLC cells treated with control, cADPR (for LLC was 50 nM, for A549 was 100 nM, 24 h) and 8-Br-cADPR (for LLC was 10 μM, for A549 was 20 μM, 24 h). **E** Western blotting was used to analysis of the expression of KEAP1 and NRF2 in A549 and LLC cells treated with an empty control PX458-plasmid (TRPM2-NC) or PX458-sgTRPM2-plasmid (sgTRPM2, TRPM2 KO). **F** A549 cells were seeded at a density of 3000 cells/well into 96-well plates treated with ML385 (10 μM) for 48 h and then cell viability was detected by using a CCK-8 assay, and the results were expressed as the OD value (*n* = 3). **G** Western blotting was used to analysis of the expression of Keap1 from LLC bearing-BALB/c-nude tumor tissues. Data were presented as mean ± SEM. ANOVA or test was used to analyze data. ns, no significant difference; *P < 0.05; **P < 0.01; ***P < 0.001, ****P < 0.001.

Analysis differences of pathways between A549 CD38 WT and CD38 KO tumor cells by Gene Ontology (GO) and Kyoto Encyclopedia of Genes and Genomes (KEGG), we identified cell cycle, cell proliferation, stress-activated MAPK signaling, cell metabolic, and other signaling pathways were changed (Fig. 6A, B). And in-depth analysis of differentially expressed genes by real-time PCR, we obtained that the mRNA expression of Nrf2-target downstream anti-oxidative genes GCLC, AKP1B10, GSTM3, and NQO1 were significantly upregulated (Fig. 6B). The KEAP1-NRF2 pathway played vital roles in regulation of cancer progression and recent studies indicated that dysfunction of KEAP1 was cooperated to drive LUAD initiation and correlated with poor survival in patients [21–24]. So we hypothesized that cADPR might via KEAP1-NRF2 signal to regulate tumor progression.

CD38 KO and MU tumor cells showed a higher expression of KEAP1 than that of WT and OE cells, while NRF2 was on the contrary (Fig. 6C). By adding cADPR and 8-Br-cADPR to co-culture tumor cells, we found that cADPR inhibited the expression of KEAP1, but promoted the expression of NRF2 in CD38 KO and MU tumor cells, while 8-Br-cADPR was on the contrary (Fig. 6D). We also verified that the changes of KEAP1 and NRF2 were corresponded with TRPM2 KO in tumor cells (Fig. 6E). In addition, our results revealed that the capability of cell proliferation was notably restrained in all groups but CD38 WT and OE tumor cells showed a more significant inhibition than CD38 KO and MU tumor cells after treated tumor cells with ML385 (Fig. 6F). And in CD38 LLC-bearing-BALB/c-nude mice tumor tissues, it was also confirmed that CD38 KO and MU-bearing-tumor tissues exhibited a higher expression of Keap1 than that of in CD38 WT and OE-bearing-tumor tissues (Fig. 6G). Altogether, these results indicated that KEAP1-NRF2 signal pathway was affected by CD38-cADPR signal.

## Discussion

Previous study clarified that PD-L1 combined with CD38 antibody could overcome PD-L1 resistance [5]. Herein, we provided a significant mechanism that the enzymatic activity of tumor expressed CD38 induced the opening of TRPM2 by generating cADPR, and resulted in an elevated concentration of cytoplasm calcium and facilitated primary tumor progression and metastasis by triggering KEAP1-NRF2 signal pathway. We also found targeting enzymatic activity of CD38 and its enzymatic product cADPR might be a promising strategy for the cancer therapy.

Indeed, in multiple myeloma, acute lymphoblastic leukemia, and other hematological cells, CD38 was abnormally upregulated and resulted in clinical manifestations of the disease include signs of organ dysfunction, along with an immunosuppressive microenvironment that finally led to hematological cells aggressiveness and poor prognosis [25, 26]. Various preclinical studies and ongoing clinical trials aimed to evaluate the efficiency of CD38 antibodies in hematological malignancies such as NK/T cell lymphoma and acute lymphoblastic leukemia [27, 28]. An updated study uncovered a phenomenon that CD38-positive macrophages used extracellular enzyme activity to inhibit the NAD^+^ levels of CD38-negative cells [8]. To date, there were no data regarding the role of CD38 in solid tumor environment. This question prompted us to investigate more in detail how the biological function of CD38 was involved in the solid tumor development, including (1) roles of CD38 ecto-enzymatic activity during tumor progression; (2) factors regulated CD38-ecto-enzymatic activity; (3) how CD38-positive cells affected the surrounding CD38-negative cells.

It is well known that CD38 is mainly a type II membrane orientation with the catalytic site facing the outside of cells, to consume the NAD^+^ and participant in the regulation of extracellular metabolic molecules, such as adenosine, nicotinamide, and cyclic ADP-ribose (cADPR) [29, 30]. Previous studies have proved that CD38 via consumed NAD^+^ to generate adenosine as a regulatory hub controls activation, redox homeostasis, pro-inflammatory effects, cell senescence, energy metabolism, and immune suppression of immune cells [31, 32]. Herein we reported that tumor expressing CD38 promoted tumor cell progression via its enzymatic product cADPR independent on NAD^+^ homeostasis and the production of adenosine. Within some specific immune cells, such as neutrophil and DC cells, cADPR was shown to regulate cell migration and trafficking through modulation of calcium influx transit [33]. We identified a role of cADPR to promote cell progression by inducing cytoplasmic calcium flux via TRPM2 channel. Consistent with our study, F. Morandi concluded that inactivation of the CD38-cADPR axis might serve as a novel therapeutic intervention in lung cancer and a recent report indicated that TRPM2 was linked with poor prognosis in patients with pancreatic ductal adenocarcinoma [34, 35]. In addition, to understand if the promotion of cADPR on tumor proliferation, and migration was a generalized phenomenon, we also demonstrated that cADPR could promote cell proliferation and migration in variety of human cell lines which existed the expression of TRPM2. Those observations might suggest that the CD38 enzymatic hydrolysis product cADPR, either from tumor cells or immune cells, might both play an important role for tumor proliferation, formation and migration independent of regulating NAD+ or adenosine in solid TME. It was well known that NRF2 acted as an oncogene, and its principal repressor KEAP1 acted as a tumor suppressor [36–38]. A recent study demonstrated that stimulating KRAS-driven LUAD cancer metastasis was induced by KEAP1 loss but NRF2 activation [24]. In the present study, we also identified CD38-cADPR exhibited a remarkable down-regulation of KEAP1 and upregulation of NRF2 in tumor cells. Furthermore, our results are relevant to the debate on the promises and perils of antioxidant supplementation, especially for lung cancer patients and for the 30% of them who harbor KEAP1/NRF2 mutations [39]. Therefore, our studies might raise the possibility that targeting CD38 enzymatic activity or downstream cADPR might be effective in patients with NRF2/KEAP1 mutation.

Notably, questions about how to incorporate the enzymatic activity of CD38 into immunotherapeutic strategies still need to be studied in prospective trials. For example, Vaisitti T elegantly showed that CD38/CD31 interactions could activate cytoplasmic calcium pathway via modulating growth and motility of CLL cells. CD38+ cells pre-incubated with anti-CD31 monoclonal antibody or 8-Br-ADPR could both abrogate CD31 binding to CD38 and block CLL chemotaxis and homing via Ca^2+^ fluxes mediated by TRPM2 [40]. Since CD38 could consume NAD^+^, NAD^+^ would be elevated by CD38 knock-out and then might stimulate SIRT1 and PARP1 to induce T cell differentiation into Th1 subsets for enhancing the anti-tumor effects [41]. Moreover, Horenstein A further broadened the horizon for the role CD38, showing that the enzymatic-product of CD38 was related to the anti-viral and pro-inflammatory response during the SARS-CoV-2 infection, independent on the CD38/NAD+ axis [42]. Apart from CD38, SARM1 were also confirmed as the additional NAD^+^-depleting enzyme that using NAD^+^ to produce cADPR, the precise functions of cADPR in those abnormal situations including tumor progression, inflammation, and injury-induced axon death need further characterizations [43, 44]. In addition, the cADPR might also be produced by CD38 expressed on the surface of immune cells such as tumor-associated macrophages (TAMs) to promote the survival of tumor cells [8].

In conclusion, our findings suggest that selectively targeting the enzymatic activity of CD38 in tumor microenvironment might represent an important strategy in treating non-hematopoietic cancers.

## Supplementary information

Supplementary figure legends

Supplementary figure 1

Supplementary figure 2

Supplementary figure 3

Supplementary figure 4

Additional Table1

Additional Table2

Additional Table3

Additional Table4

Additional Table5

## Data Availability

Specific details will be provided if request.
