# Supplementary material for: The intrinsic role and mechanism of tumor expressed-CD38 on lung adenocarcinoma progression
Source: Cell Death Dis. 2021 Jul 5;12(7):680. doi: 10.1038/s41419-021-03968-2 (PMC8256983; doi:10.1038/s41419-021-03968-2)
Supplement: Supplementary file 1 — Supplementary figure legends [file 41419_2021_3968_MOESM1_ESM.docx]

**Supplementary figure legends**

**Supplementary figure 1:** FACS analysis was used to measure the expression of CD38 and CD157 on cells purified pleural effusion, which originated from lung adenocarcinoma patients (n=3).

**Supplementary figure 2: The enzymatic activity of CD38 was vital for cell survival. (A)** The expression of CD38 A549 and LLC tumor cells were detected by FACS. **(B).** Mutagenesis of the human and mouse CD38 MU and OE DNA sequence encoding the enzyme site residue (for LLC was constructed LLC-CD38-Cysteine123+Cysteine205 enzyme sites mutant plasmids; for A549 was constructed A549-CD38-glutamate146+glutamate226 enzyme sites mutant plasmids) was confirmed by sequencing analysis. **(C).** Representative immunofluorescence image for CD38 (green) and nucleus (DAPI) in A549 tumor cells were performed. **(D).** LLC and LLC-T2 cells were separately implanted into immunocompetent C57BL/6 mouse models (2×10^5^ cells per mouse). Tumors were measured every two days at day of 7. The tumor growth curve was shown with tumor sizes (n=5). **(E).** The expression of CD38 was detected by in multiple tumor cell lines across human and murine species. **(F).** FACS was used to analysis of % MDSCs (upper), macrophages (middle) and CD3 T+ tumor infiltrating lymphocytes (TILs, downer) cells from primary tumors in immunocompetent C57BL/6 mice (n=4). **(G).** Growth of subcutaneous LLC tumor cells (2×10^5^ cells per mouse) in CD38-deficiency mice (n=5) (left). Tumors were measured every two days beginning on day 7 after the tumor cell implantation. Mice were sacrificed at day of 17 after injection. The tumor mass was shown with tumor sizes (right). **(H).** FACS was used to analysis of % CD3 T+ tumor infiltrating lymphocytes (TILs) cells from primary tumors in CD38-deficiency mice (n=4). **(I).** A549 (1×10^5^ cells per well) cells were seeded at the upper-chambers for 48h and then the numbers of migrated cells that adhered to the lower surface of the trans-well chambers were counted under an inverted microscope randomly chosen visual fields per well within the area (n=3). **(J).** Representative FACS plots was used to perform the apoptotic rate of A549. In vitro apoptotic cells data was representative of three independent experiments. ANOVA or t test was used to analyze the data. The data were presented as mean±SEM, ns, no significant difference; *, P < 0.05; **, P < 0.01; ***, P < 0.001, ****, P < 0.0001.

**Supplementary figure 3: CD38 used its hydrolyze product cADPR to promote the capability of cell survival. (A).** The supernatants and cell lysis substance from A549 and LLC tumor cells were subjected to HPLC assay to measure NAD+ and adenosine (n=2). **(B).** A549 (1×10^5^ cells per well) cells were seeded at the upper-chambers adding with NMN (10μM, left) or adenosine (5μM, right) for 24h and then the numbers of migrated cells that adhered to the lower surface of the trans-well chambers were counted under an inverted microscope randomly chosen visual fields per well within the area (n=3). **(C)** A549 cells were seeded at a density of 3,000 cells/well into 96-well plates treated with NMN (20μM, left) or adenosine (5μM, right) for 48h and then cell viability was detected using a CCK-8 assay, and the results were expressed as the OD value (n=3). **(D).** LLC (1×10^5^ cells per well) cells were seeded at the upper-chambers adding with 8-Br-cADPR (10μM) for 24h and then the numbers of migrated cells that adhered to the lower surface of the trans-well chambers were counted under an inverted microscope randomly chosen visual fields per well within the area (n=3). **(E).** LLC cells were cultured at a density of 500 cells/well into 24-well plates treated with cADPR (50nM) for 7 days and then the number of colony formation was visualized by staining with 0.1% crystal violet, and the results were shown as the number of positive area randomly (n=3). **(F).** FACS was used to analysis of % MDSCs (upper), macrophages (middle) and CD3 T+ tumor infiltrating lymphocytes (TILs, downer) cells from primary tumors treated with 8-Br-cADPR (0.5mg/kg) or PBS as control in immunocompetent C57BL/6 mice (n=4). **(G).** FACS was used to analysis of % MDSCs (upper), macrophages (middle) and CD3+ T tumor infiltrating lymphocytes (TILs, downer) cells from primary tumors treated with 78C (10mg/kg) or PBS as control in immunocompetent C57BL/6 mice (n=3). Data were shown as mean ± SEM. ANOVA or t test was used to analyze the data. ns, no significant difference; *, P < 0.05; **, P < 0.01; ***, P < 0.001.

**Supplementary figure 4: cADPR induced the Ca^2+^ influx into intracellular via TRPM2 channel.** **(A).** Western blotting was used to detect TRPM2 protein LLC and A549 tumor cells, GAPDH as a loading control. **(B).** The expression of TRPM2 were detected by western blotting in A549 cells transfected with a scramble control (TRPM2-con) or siRNA against TRPM2 (siTRPM2). **(C).** TRPM2 expression were detected by western blotting in A549 (upper) and LLC (downer) cell lines transfected with an empty control PX458-plasmid (TRPM2-NC) or PX458-sgTRPM2-plasmid (sgTRPM2, TRPM2 KO). **(D).** LLC (1.5×10^5^ cells per well) cells treated with control (TRPM2-con) or a small molecular inhibitor (2-APB, 100μM) were seeded at the upper-chambers adding for 24h and then the numbers of migrated cells that adhered to the lower surface of the trans-well chambers were counted under an inverted microscope randomly chosen visual fields per well within the area (n=3). **(E).** Western blotting assay was used to detect TRPM2 protein in A549, MBA-MD231, HCC827, HCT116, BEL7402, HepG2, PANC-1, SW480 and PanCO2 tumor cells. **(F).** PanCO2 (1×10^5^ cells per well) tumor cells treated with cADPR (100nM), 8-Br-cADPR (20μM) or a small molecular inhibitor (ACA,50μM) were seeded at the upper-chambers adding for 24h and then the numbers of migrated cells that adhered to the lower surface of the trans-well chambers were counted under an inverted microscope randomly chosen visual fields per well within the area (n=3). **(G).** MBA-MD231, HCC827, HCT116, BEL7402, PANC-1 and PanCO2 tumor cells treated with 8-Br-cADPR (20μM) were seeded at a density of 3000 cells/well into 96-well plates for 48h and then cell viability was detected using a CCK-8 assay, and the results were expressed as the OD value (n=3). **(H).** HCC827, HCT116, PANC-1 and PanCO2 tumor cells treated with cADPR (100nM) were seeded at a density of 3000 cells/well into 96-well plates for 48h and then cell viability was detected using a CCK-8 assay, and the results were expressed as the OD value (n=3). Data were presented as mean±SEM. ANOVA or test was used to analyze data. ns, no significant difference; *, P < 0.05; **, P < 0.01; ***, P < 0.001, ****, P < 0.0001.
