# Supplementary figures and images for: The intrinsic role and mechanism of tumor expressed-CD38 on lung adenocarcinoma progression
Source: Cell Death Dis. 2021 Jul 5;12(7):680. doi: 10.1038/s41419-021-03968-2 (PMC8256983; doi:10.1038/s41419-021-03968-2)

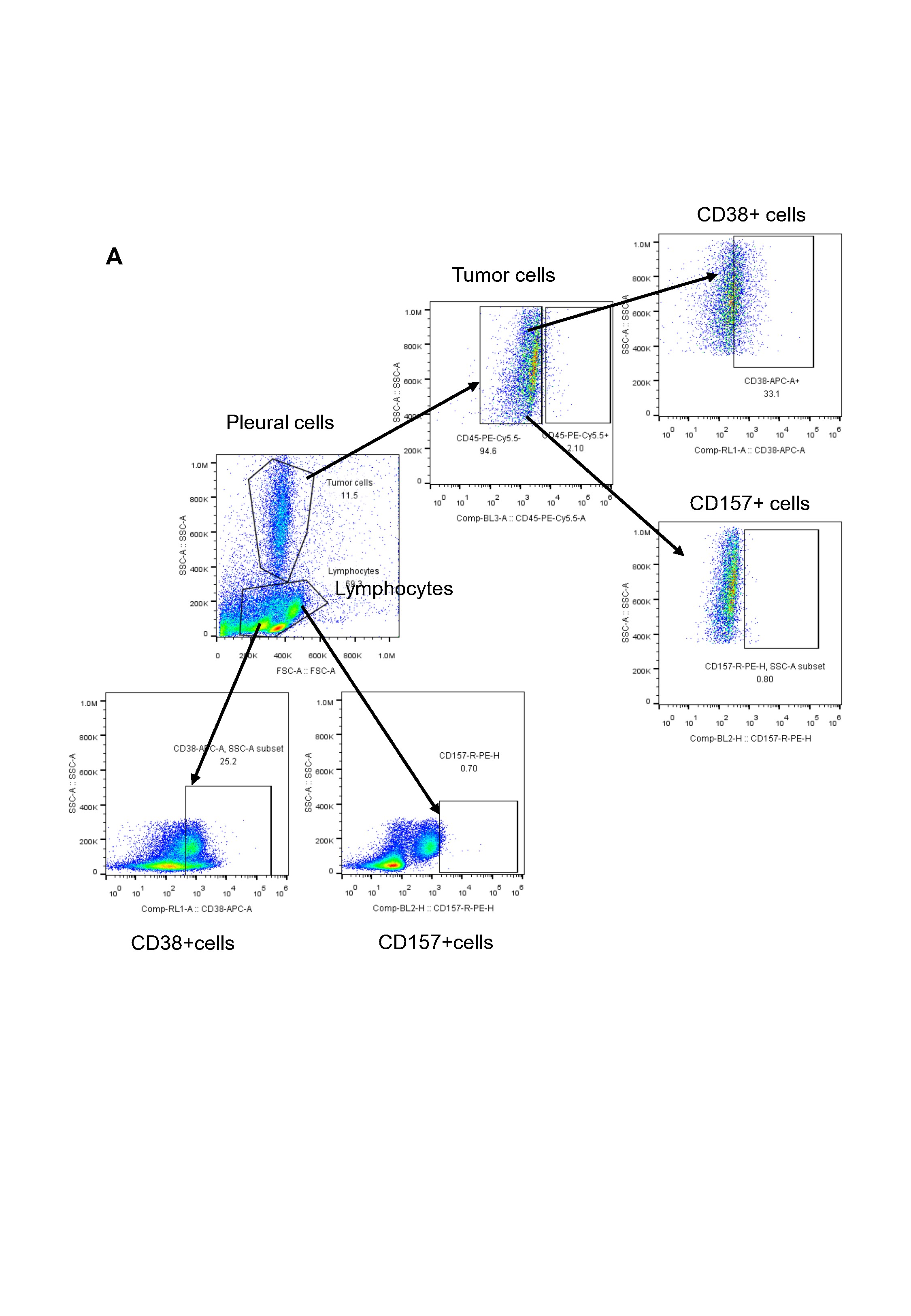

Supplement: Supplementary file 2 — Supplementary figure 1 [file 41419_2021_3968_MOESM2_ESM.tif]

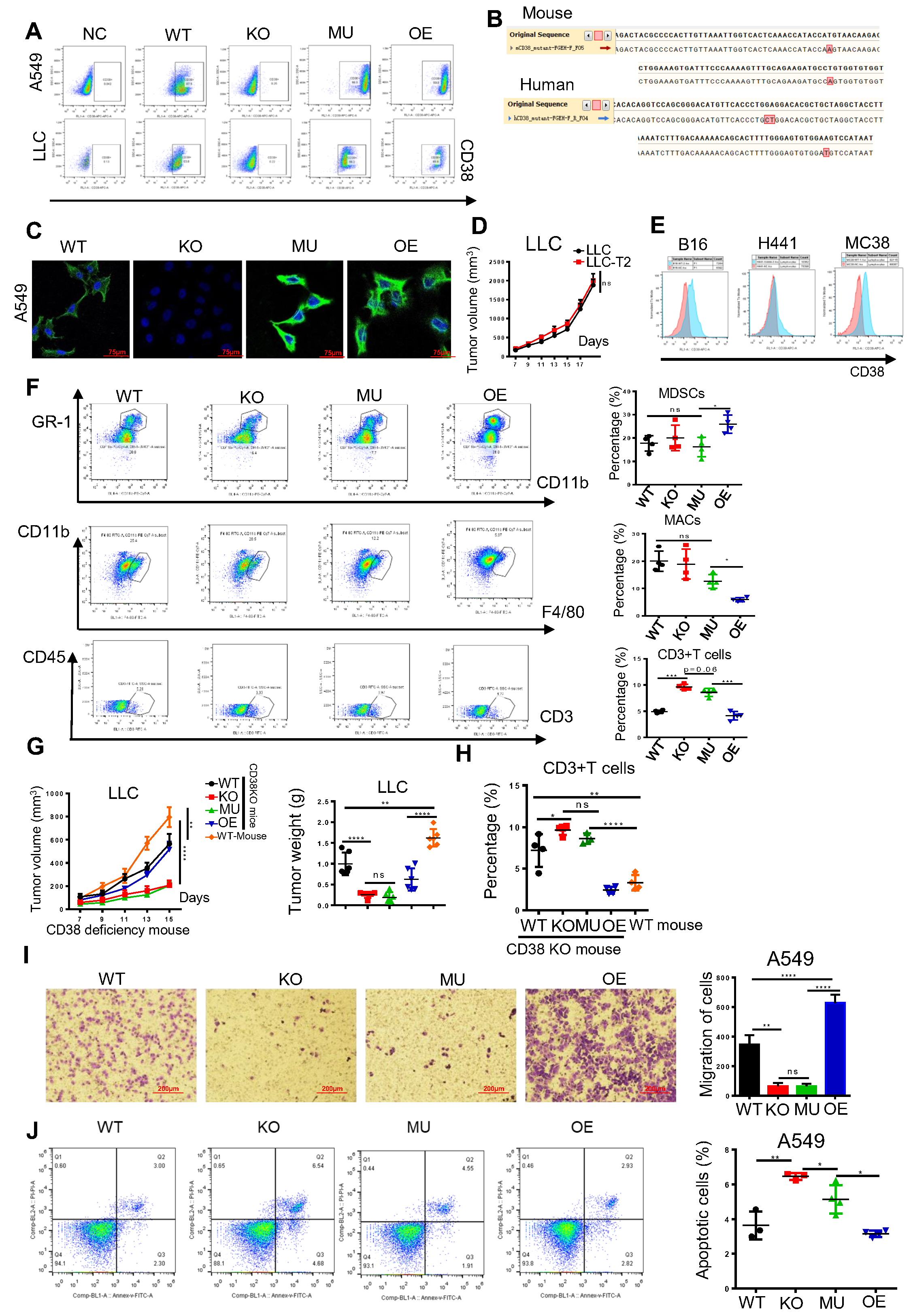

Supplement: Supplementary file 3 — Supplementary figure 2 [file 41419_2021_3968_MOESM3_ESM.tif]

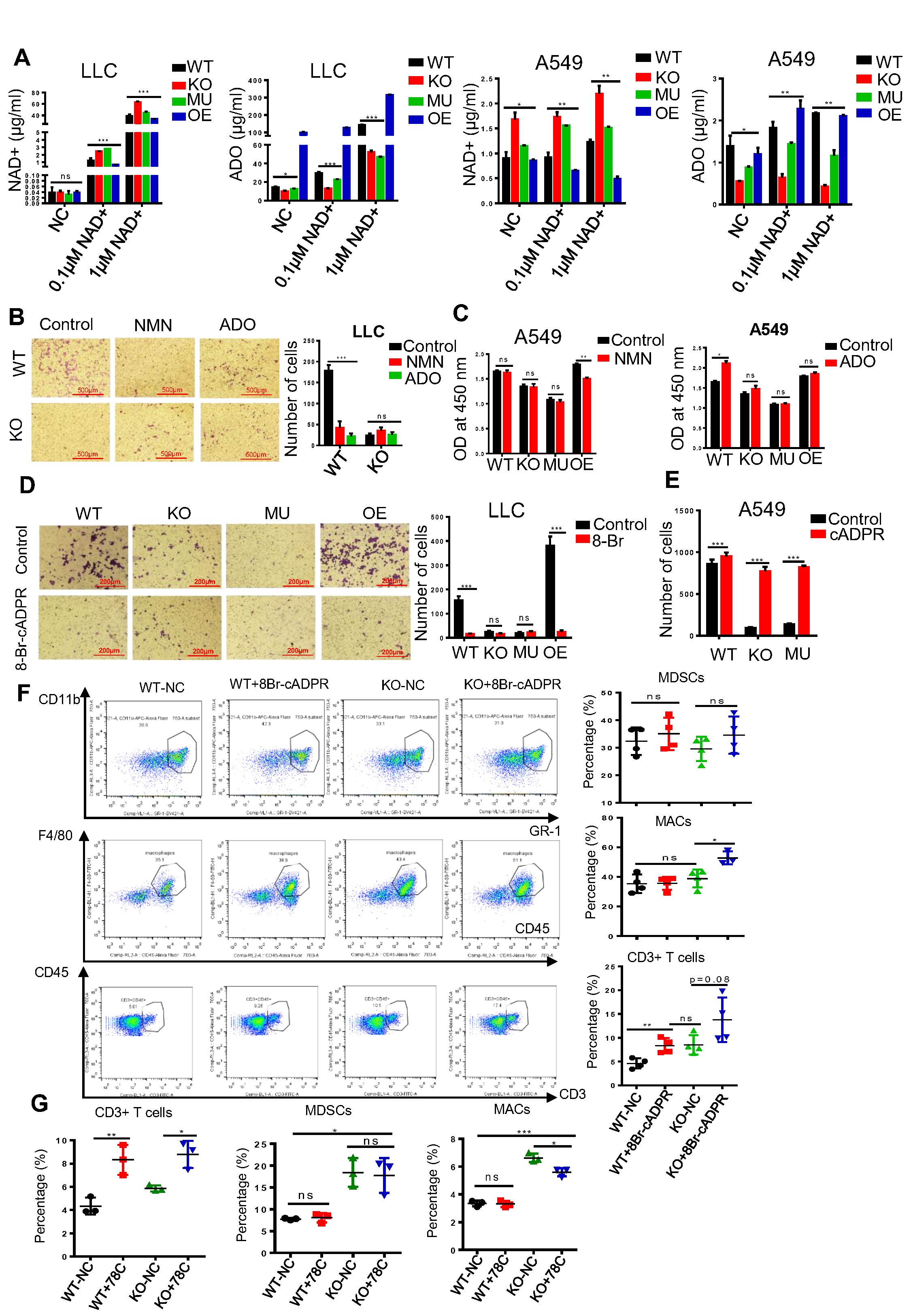

Supplement: Supplementary file 4 — Supplementary figure 3 [file 41419_2021_3968_MOESM4_ESM.tif]

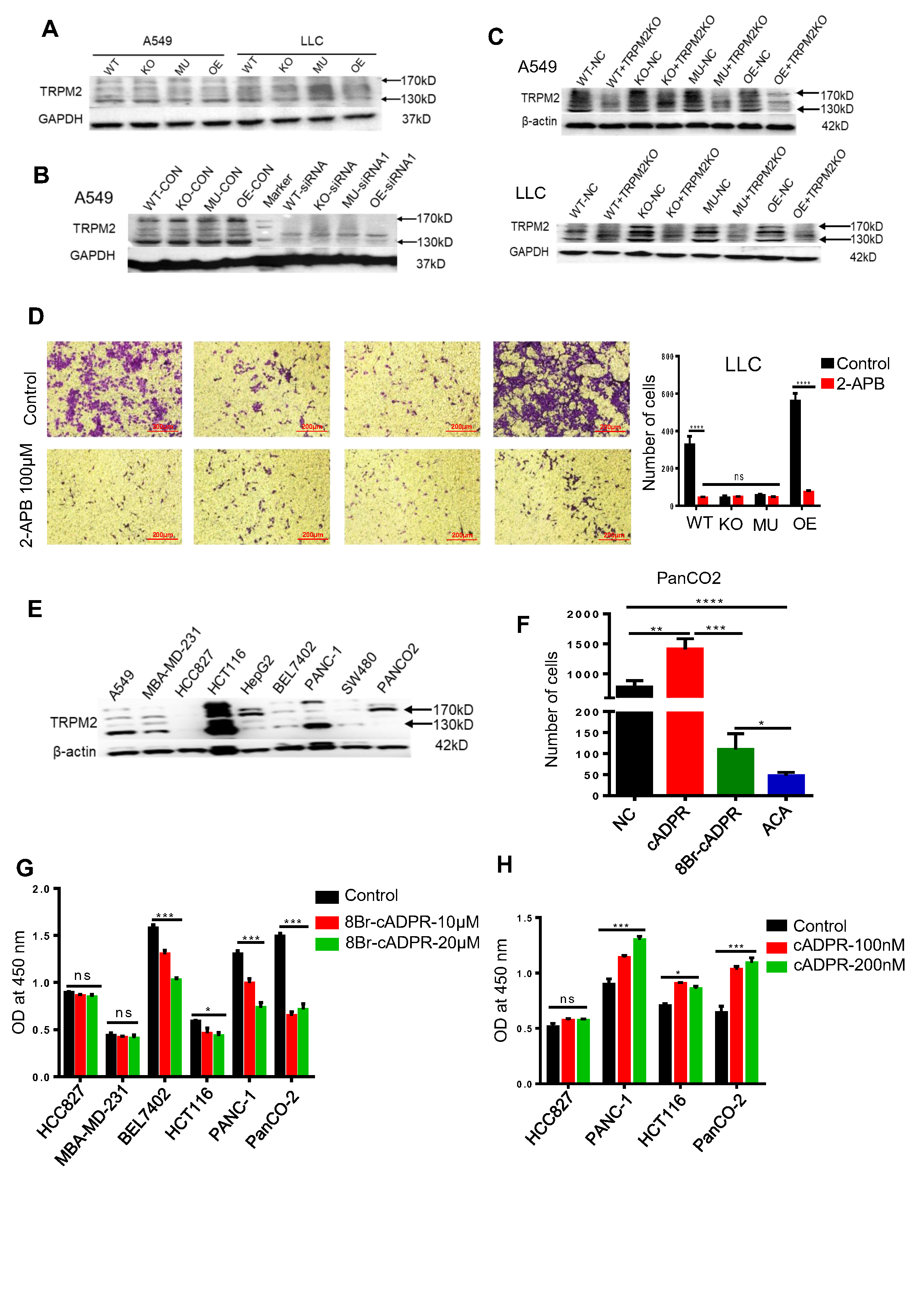

Supplement: Supplementary file 5 — Supplementary figure 4 [file 41419_2021_3968_MOESM5_ESM.tif]
