# Supplementary material for: The intrinsic role and mechanism of tumor expressed-CD38 on lung adenocarcinoma progression
Source: Cell Death Dis. 2021 Jul 5;12(7):680. doi: 10.1038/s41419-021-03968-2 (PMC8256983; doi:10.1038/s41419-021-03968-2)
Supplement: Supplementary file 6 — Additional Table1 [file 41419_2021_3968_MOESM6_ESM.docx]

Additional table 1: small molecule drugs or inhibitors

| Small drugs | Source | Identifier | concentration |
| --- | --- | --- | --- |
| NAD+ | MCE | HY-B0445 | 0.5μM and 1μM |
| NMN | MCE | HY-F0004 | 10μM and 20μM |
| Adenosine | MCE | HY-B0228 | 5μM and 10μM |
| cADPR | MCE | HY-N7395 | 50nM and 100nM |
| 8-Br-cADPR | Santa- cruz | sc-201514A | 10μM and 20μM |
| 2-APB | Sigma-Aldrich | 100065 | 100μM |
| ACA | Sigma-Aldrich | 104550 | 10μM |
| ML385 | MCE | HY-100523 | 10μM |
| MG132 | Sigma-Aldrich | M8699 | 5μM |
