# Supplementary material for: The intrinsic role and mechanism of tumor expressed-CD38 on lung adenocarcinoma progression
Source: Cell Death Dis. 2021 Jul 5;12(7):680. doi: 10.1038/s41419-021-03968-2 (PMC8256983; doi:10.1038/s41419-021-03968-2)
Supplement: Supplementary file 7 — Additional Table2 [file 41419_2021_3968_MOESM7_ESM.docx]

Additional table 2: siRNA for TRPM2 knock-down

| Name | Sequence |
| --- | --- |
| Mouse-siTRPM2-1 | 5′‐UAAGCGUUCAUGCUCUUCUGCCAGC‐3′ |
| Mouse-siTRPM2-2 | 5′-GUCUCGGACAUCACUAUCUTT-3′ |
| Mouse-siTRPM2-3 | 5′-AACUUUUCCAGCUUAGUCCUC-3′ |
| Human-siTRPM2-1 | 5′-UCUUCUUGAUGUUUUCAGGAA-3′ |
| Human-siTRPM2-2 | 5′-UUCAUGUUGAAGUUCUUGGCC-3′ |
| Human-siTRPM2-3 | 5′-ACAAACUCAGGCUUGUUGGAG-3′ |
