# Supplementary material for: The intrinsic role and mechanism of tumor expressed-CD38 on lung adenocarcinoma progression
Source: Cell Death Dis. 2021 Jul 5;12(7):680. doi: 10.1038/s41419-021-03968-2 (PMC8256983; doi:10.1038/s41419-021-03968-2)
Supplement: Supplementary file 8 — Additional Table3 [file 41419_2021_3968_MOESM8_ESM.docx]

Additional table 3: sgRNA for TRPM2 or CD38 knock-out

| Name | Sequence |
| --- | --- |
| Mouse-sgTRPM2-1 | 5′‐GCCAGTTCTTCTCCGGTCCA‐3′ |
| Mouse-sgTRPM2-2 | 5′- GTATTGCTTCGTCGGAGATTG -3′ |
| Human-sgTRPM2-1 | 5′- GAGGAAAGCTGGCTCGGAGC -3′ |
| Human-sgTRPM2-2 | 5′-GAACTCACCGCGTGACCATG-3′ |
| Mouse-sgCD38-1 | 5′‐ GTGAATTTAGCCAGGTGTCTG‐ 3′ |
| Mouse-sgCD38-2 | 5′- GCGATCT GGGCTTTCCTAGAG-3′ |
| Human-sgCD38-1 | 5′- GCGCTTTCCCGAGACCGTCC -3′ |
| Human-sgCD38-2 | 5′-GTGTACTTGACGCATCGCGCC-3′ |
