# Supplementary material for: The intrinsic role and mechanism of tumor expressed-CD38 on lung adenocarcinoma progression
Source: Cell Death Dis. 2021 Jul 5;12(7):680. doi: 10.1038/s41419-021-03968-2 (PMC8256983; doi:10.1038/s41419-021-03968-2)
Supplement: Supplementary file 9 — Additional Table4 [file 41419_2021_3968_MOESM9_ESM.docx]

Additional table 4: primary flow cytometry antibodies used in our project

| antibodies | Source | Identifier |
| --- | --- | --- |
| Alexa Fluor® 700 Rat Anti-Mouse CD45 | BD | 560510 |
| FITC anti-mouse CD3 Antibody | BioLegend | 100204 |
| PE/Cy7 anti-mouse CD4 Antibody | BioLegend | 100422 |
| APC-CD8b Monoclonal Antibody | eBioscience | 11008385 |
| PerCP-Cy™5.5 Rat Anti-CD11b | BD | 550993 |
| Brilliant Violet 421™ anti-mouse GR-1 | BioLegend | 108433 |
| FITC anti-mouse F4/80 Antibody | BioLegend | 123108 |
| CD157 Monoclonal Antibody, PE | Invitrogen | 12-1579-42 |
