# Supplementary material for: The intrinsic role and mechanism of tumor expressed-CD38 on lung adenocarcinoma progression
Source: Cell Death Dis. 2021 Jul 5;12(7):680. doi: 10.1038/s41419-021-03968-2 (PMC8256983; doi:10.1038/s41419-021-03968-2)
Supplement: Supplementary file 10 — Additional Table5 [file 41419_2021_3968_MOESM10_ESM.docx]

Additional table 5: primers for qRT-PCR

| Name | Sequence |
| --- | --- |
| GAPDH-F | 5′‐GGCATGGACTGTGGTCATGAG‐3′ |
| GAPDH-R | 5′‐TGCACCACCAACTGCTTAGC‐3′ |
| GCLC-F | 5′-AAACCCAAACCATCCTACCC-3′ |
| GCLC-R | 5′-GCATGTTGGCCTCAACTGTA-3′ |
| NQO1-F | 5′-ATGTATGACAAAGGACCCTTCC-3′ |
| NQO1-R | 5′-TCCCTTGCAGAGAGTACATGG-3′ |
| GSTM3-F | 5′-CCGCACACAACTGATAAGGC-3′ |
| GSTM3-R | 5′-CCAGGCACTTGGGGTCAAAT-3′ |
| AKR1B10-F | 5′-ATCACCGTTACGGCCTACAG-3′ |
| AKR1B10-R | 5′-TGTCACAGACTTGGGGATGA-3′ |
